# Predictions of Wear Performances of AlSi7Mg0.6 Cast Aluminum Alloy Under Different Displacement and Applied Load

**DOI:** 10.3390/ma19040752

**Published:** 2026-02-14

**Authors:** Guoqing Gu, Yun Ma, Fei Du, Aiguo Zhao

**Affiliations:** 1 School of Civil Engineering, Yancheng Institute of Technology, Yancheng 224051, China; gqgu@ycit.edu.cn (G.G.); myedu0510@163.com (Y.M.); 2 College of Civil Engineering, Nanjing Tech University, Nanjing 211816, China; njtech_df@163.com

**Keywords:** AlSi7Mg0.6 aluminum alloy, wear performance, UMESHMOTION subroutine, wear simulation

## Abstract

AlSi7Mg0.6 aluminum alloy is widely adopted in many industrial fields due to its favorable mechanical properties and lightweight merits. In the catenary system of high-speed railways, AlSi7Mg0.6 aluminum alloy is adopted as the substrate of the positioning hook and positioning support, which exhibit abnormal wear in some railways. Thus, it is very important to reveal the underlying wear characteristics and discover the key factors involved. In this study, the influences of displacement (0.5 mm, 1.5 mm, and 3.0 mm) and applied load (20 N, 50 N, 100 N, and 200 N) on the wear performance of AlSi7Mg0.6 aluminum alloy are investigated experimentally and numerically. Wear experiments are time-consuming and costly, but the finite element method (FEM) can effectively solve this problem. A UMESHMOTION user-defined subroutine integrated with an ABAQUS Arbitrary Lagrangian–Eulerian (ALE) adaptive mesh technique was developed to simulate the wear evolution process of the aluminum alloy under varying displacements and applied loads. The results indicate that the wear evolution process of AlSi7Mg0.6 aluminum alloy can be effectively simulated using the UMESHMOTION subroutine. The maximum wear depth (MWD) from the FEM deviates from the experimental results by no more than 10%, and the deviation is smaller than the experimental values. The largest deviation occurs when the displacement is 3.0 mm and the applied load is 100 N, where the discrepancy reaches 7.53%. The wear volume (WV) obtained from the FEM shows a deviation of less than 20% compared to experimental results. For the case with a displacement of 0.5 mm, the numerical results underestimate the wear volume, while for the case with displacements of 1.5 mm and 3.0 mm, the numerical results overestimate the wear volume. The largest deviation in this case occurs for the case with a displacement of 3.0 mm and applied loading of 100 N, with a discrepancy of 16.33%.

## 1. Introduction

AlSi7Mg0.6 aluminum alloy is a lightweight material known for its excellent casting properties, superior mechanical characteristics and relatively low density, and it has been widely used in various engineering and industrial fields. Particularly in high-load and high-performance sectors such as the automotive, aerospace and railway sectors, AlSi7Mg0.6 alloy has become a critical structural material due to its outstanding corrosion resistance, high strength-to-weight ratio and favorable machinability. In the high-speed railway catenary system, AlSi7Mg0.6 alloy is widely employed in key components such as positioning hooks and support structures. These components are subjected to substantial dynamic loads, frequent frictional forces and extreme environmental corrosion conditions. Therefore, wear-related issues become particularly pronounced over prolonged use. The intensification of wear not only compromises the system’s safety performance, but also significantly reduces the service life of key components.

Investigating the friction and wear behavior of AlSi7Mg0.6 is crucial due to its widespread industrial applications. According to the perspectives of Archard and Hirst [1], the sliding wear behavior of aluminum alloys can be classified into two stages: “mild” and “severe” wear. Venkataraman et al. [2] studied the wear performance of 7075 aluminum and Al-SiC composites, highlighting the role of the mechanical mixed layer (MML) in wear behaviors. Elleuch et al. [3] identified a critical displacement in A357 aluminum alloy, beyond which wear transitions to a high-wear state. Kim et al. [4] demonstrated that higher sliding speeds reduced the friction coefficient in aluminum alloys. Yang et al. [5] observed that Sr modifiers in A357 alloys formed a stable mixed layer and reduced the wear rate. Mondal et al. [6] investigated the effect of load on the wear behavior of Al-Zn-Mg alloys and their particulate-reinforced composites. The results indicated that the wear rate increased with the applied pressure, and the composite materials exhibited superior wear resistance compared to the alloys. Chandrashekharaiah et al. [7] studied the dry-sliding tribological properties of grain-refined and modified eutectic Al-12Si alloys and observed adhesive wear on the wear surface. Chen et al. [8] studied the wear behavior of aluminum conductor steel-reinforced cables that had been in operation for over 26 years, suggesting that adhesive and abrasive wear were the primarily failure modes. Lakshmikanthan et al. [9] concluded that larger SiC particles in A357 composites improved the tensile strength and wear resistance efficiently. Çolak et al. [10] showed that Al5Ti1B grain refiners improved the wear resistance of A357 alloys by reducing friction. Lorenzetti et al. [11] found that LPBF processing could refine the microstructure of AlSi7Mg0.6 and reduce wear rates. Tan et al. [12] demonstrated that A357/SiC composites fabricated by friction stir processing exhibited excellent wear and friction performances. Rong et al. [13] prepared AlSi10Mg alloy specimens by spark plasma sintering and found that the sintering temperature had a significant effect on grain size, as well as on the eutectic silicon size and the wear and corrosion properties after heat treatment. Xiao et al. [14] fabricated a dual-phase microstructure consisting of an aluminum-matrix composite and cast ZL101 aluminum alloy by friction stir lap welding (FSLW). The results demonstrated that the interfacial bonding strength exceeded the load-bearing capacity of the alloy, while the wear resistance of the composite was significantly improved. Patarić et al. [15] investigated the role of silicon in modifying the Al-Si-Al alloy phase diagram. Their results confirmed that a higher Si content improves feeding capability by narrowing the solidification range and reducing the risk of porosity. An increase in silicon content was shown to enhance feeding ability and castability by narrowing the solidification interval and promoting more uniform solidification in Al-Si-Mg alloys. Zhang et al. [16] performed simulations of AlSi10Mg alloy using a crystal plasticity finite element method and proposed a stress concentration factor characterization approach that simultaneously considers pore size, morphology, and spatial distribution. The simulation results indicated a competitive mechanism between pores and grains; with respect to the influence of aspect ratio on stress concentration, a pronounced increase in stress concentration was observed as the aspect ratio decreased along the loading direction.

Most studies employ the Archard wear model to calculate wear depth [17,18,19], updating the surface shape at each time increment and using the UMESHMOTION subroutine to control mesh node movement in the normal direction. Söderberg et al. [20] applied Archard’s theory to simulate the wear of brake disks with a simplified 3D finite element model in ANSYS. Brouzoulis et al. [21] proposed a plasticity-based wear simulation model based on real wheel–rail contact data. Rezaei et al. [22] studied the wear of rotating shafts and sleeves, wherein adaptive mesh techniques and a hybrid Lagrangian–Eulerian algorithm were adopted to simulate contact conditions. Cruzado et al. [23] established a finite element model with ABAQUS to study the contact wear of steel wires, where the impact of mesh size, cycle numbers and jump cycles on wear depth were analyzed with UMESHMOTION subroutines. Martínez et al. [24] developed a comprehensive numerical model for polymer–metal friction and wear, employing ALE meshing to maintain mesh quality in ABAQUS. Shen et al. [25] developed a thermal-wear simulation program based on thermomechanical finite element analysis to assess the wear performance of self-lubricating fabric-lined spherical sliding bearings. Bortoleto et al. [26] built a pin-disk friction model in ABAQUS based on the Archard equation and UMESHMOTION subroutine, suggesting that simulated wear would exceed experimental results due to overestimated wear coefficients. Lengiewicz et al. [27] developed a numerical model for pin-disk wear based on the Archard model, calculating contact pressure without considering elastic deformation, and validated the model with a 3D finite element approach. Wang et al. [28] developed a model to study the fretting fatigue of steel cables, incorporating wear coefficients, energy dissipation and life prediction, wherein the effects of corrosive media on wear were also examined. Ashraf et al. [29] proposed a wear algorithm model for predicting the sliding wear behavior of composite cobalt-based alloys. Lian et al. [30] conducted numerical wear simulations on hard alloy disks with three different textures, where the hole-texture disk exhibited the least wear depth. Combining the models of Hobson and Archard, Akama et al. [31] studied the crack propagation and wear behaviors during rolling contact fatigue of wheel treads, yielding highly applicable results. Wang et al. [32] proposed a finite-element-based approach to investigate the influence of fretting wear on fatigue crack propagation. In their framework, the wear evolution was predicted using the Archard wear model, while the classical Paris law was modified by incorporating wear-rate effects to describe fatigue crack growth. Pan et al. [33] developed a three-dimensional fretting wear model for a ball-on-plane contact configuration by coupling the finite element method with an enhanced energy-based wear formulation. The reliability of the proposed model was validated through comparisons with Hertzian contact theory and corresponding fretting wear experiments. Wei et al. [34] conducted systematic fretting wear tests on M50 bearing steel to determine critical parameters, including the fretting wear coefficient and wear rate. By implementing the UMESHMOTION subroutine, they established a verified three-dimensional fretting wear model based on energy dissipation. Men et al. [35] further extended the recently introduced local variable wear coefficient (LVWC) model by refining its functional expression and applying it to simulate fretting wear behavior of Ti-6Al-4V alloy under full-contact conditions. Chen et al. [36] proposed a novel numerical strategy to simulate the generation and dynamic evolution of wear debris by integrating a material conversion technique with an energy-dissipation-based wear model.

In the field of wear depth simulation and analysis, current research primarily focuses on using the birth–death element method in ANSYS and the ALE adaptive mesh technique in ABAQUS to dynamically update the wear surface. The birth–death element method achieves material removal by “killing” elements that reach critical wear depth, making it relatively straightforward. This approach essentially removes material elements, potentially causing drastic changes in the model’s stiffness matrix and deteriorating convergence. It fails to accurately reflect the smooth surface morphology after wear and struggles to precisely control the geometric shape of material removal. Consequently, its simulation accuracy is limited for problems involving large slip or complex contact. Methods like the extended finite element method (XFEM) and crystal plasticity finite element method (CPFEM) can account for crack propagation and material anisotropy at finer scales, but their computational cost is extremely high. They are thus primarily used for mechanism studies rather than engineering-scale wear prediction. When selecting the numerical simulation method for this study, we primarily considered the characteristics of the problem and the required accuracy. The reciprocating sliding contact between AlSi7Mg0.6 aluminum alloy and steel balls under varying displacement and load constitutes a complex process involving geometric nonlinearity and material removal. The classical Archard wear model, valued for its simplicity, clear physical interpretation of parameters, and ease of experimental calibration, is widely adopted for quantifying engineering wear and thus serves as the core theoretical framework for this simulation. To achieve dynamic evolution of the wear process in finite element analysis, a technique capable of high-fidelity surface topography updates is essential. Adaptive mesh refinement (ALE) enables the mesh to remain independent of material motion, effectively preventing severe mesh distortion caused by surface node displacement during sliding. This ensures computational stability and accuracy under conditions of large slip.

Based on the wear characteristics obtained from wear experiments on AlSi7Mg0.6 aluminum alloy under different displacements and applied loads, the average wear volume formula was employed to calculate twelve corresponding wear coefficients. Most existing studies neglect the influence of different loading modes on the wear coefficient. In this study, a UMESHMOTION subroutine was developed based on Archard’s wear theory, in which each loading mode is associated with a specific wear coefficient. A finite element wear model of a GCr15 steel ball sliding against an AlSi7Mg0.6 aluminum alloy plate was established in Abaqus by incorporating the ALE adaptive meshing technique, enabling numerical prediction of wear characteristics under various experimental loading conditions. Then, the numerical results were compared with those of experimental results under different displacements and loads, which was adopted to verify the accuracy of this methodology.

## 2. Materials and Methods

### 2.1. Materials

**Figure 1:**
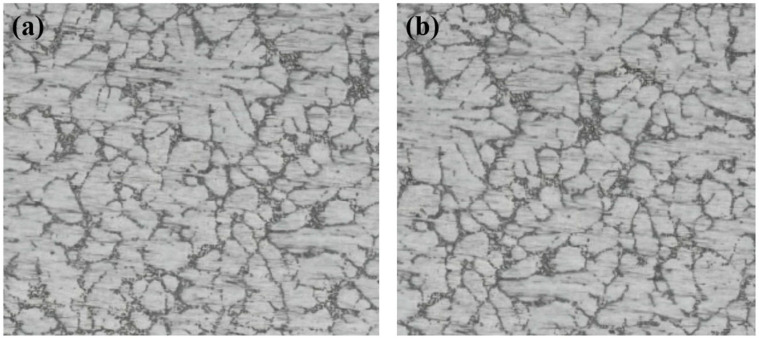
Optical microstructures of the studied AlSi7Mg0.6 alloy [37]. (**a**) Section 1. (**b**) Section 2.

The material used in the experiment is AlSi7Mg0.6 aluminum alloy, and the chemical composition is shown in Table 1. The materials were produced by Shangxi Qingye Special Materials Co., Ltd.(Xi’an, China). The microstructures of this alloy are shown in Figure 1, primarily consisting of coarse microstructures and distinct dendritic structures. The test samples are aluminum blocks with dimensions of 20 mm × 15 mm × 4 mm, with a total of four samples prepared. Prior to wear tests, all samples were polished using sandpapers with grits of 800, 1500, and 2000, respectively, and then they were cleaned with ethanol in an ultrasonic bath. The corresponding steel balls were made from GCr15 material, tempered at 180 °C, with a diameter of 10 mm.

### 2.2. Experimental Method

**Figure 2:**
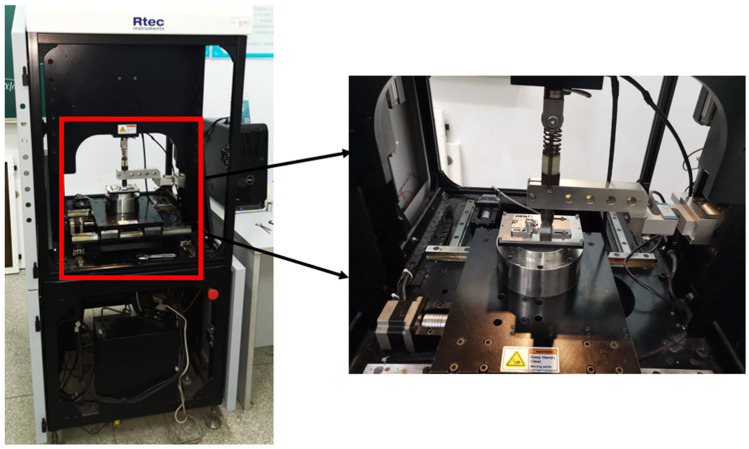
Reciprocating friction wear testing machine [37].

The experiments were conducted in dry air at room temperature (25 °C) using a reciprocating friction wear testing machine (RTEC, San Jose, CA, USA), as shown in Figure 2. During experiments, the GCr15 steel ball was fixed in the upper position, and the applied load was controlled using a spring-loaded holder. To reduce the impact of wear scars, the steel ball was replaced with a new one after each test condition. The AlSi7Mg0.6 aluminum alloy samples were secured onto the platform using fixtures, and the platform reciprocated during the test. The platform speed was set to 10 mm/s, and the load duration was set to 20 min, remaining constant throughout the entire experiment. To study the effect of maximum sliding amplitude, the displacements used in the experiment were 0.5 mm, 1.5 mm and 3.0 mm. A displacement of 0.5 mm was used to simulate rolling contact friction, while the other two displacements were used to simulate sliding contact friction. To study the effect of applied load, the applied loads were 20 N, 50 N, 100 N and 200 N.

## 3. Experimental Results

### 3.1. Friction Coefficient

**Figure 3:**
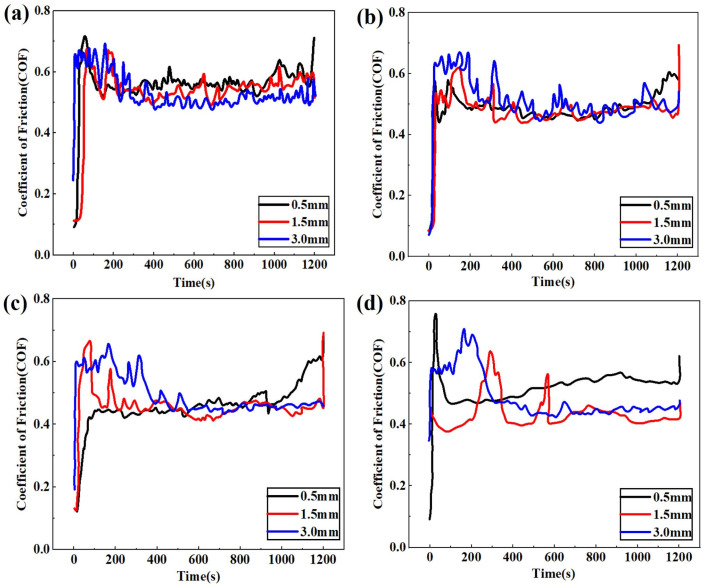
Instantaneous coefficient of friction (CoF) between AlSi7Mg0.6 and GCr15 steel ball under different displacements and applied loads [37]. (**a**) 20 N. (**b**) 50 N. (**c**) 100 N. (**d**) 200 N.

The friction coefficient measured during the experiment varies with wear time, as shown in Figure 3. Observations reveal that under identical applied loads of 20 N, 50 N, and 100 N, the friction coefficient fluctuates near the same stable value despite differing displacements. However, it is particularly noteworthy that at a load of 200 N, the friction coefficient during the steady-state phase at a displacement of 0.5 mm is higher than that at displacements of 1.5 mm and 3.0 mm. Considering that the friction coefficient exhibits rapid growth only during the initial stage, corresponding to fewer than 100 wear cycles, the friction coefficients selected for the ABAQUS 2024) simulation analysis of the contact between the GCr15 steel ball and the AlSi7Mg0.6 aluminum alloy under 20 N, 50 N, and 100 N loads correspond to the steady-state phase of the fitted experimental data, namely 0.51, 0.46, and 0.42, respectively. At a load of 200 N, a friction coefficient of 0.51 was selected for a displacement of 0.5 mm, while a coefficient of 0.42 was chosen for displacements of 1.5 mm and 3.0 mm.

### 3.2. Wear Scars

**Figure 4:**
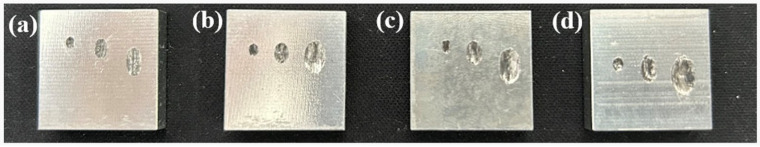
AlSi7Mg0.6 samples after wear testing. The wear scars, from left to right, correspond to displacements of 0.5 mm, 1.5 mm, and 3.0 mm, respectively. (**a**) 20 N. (**b**) 50 N. (**c**) 100 N. (**d**) 200 N.

During the measurements, each wear scar was repeated three times. The samples after testing are shown in Figure 4. For each sample, the same load was applied but with different displacements. On each sample, the wear scars from left to right correspond to displacements of 0.5 mm, 1.5 mm and 3.0 mm. It can be clearly seen that an increase in displacement directly leads to an enlargement of wear scars or an increase in the worn volume.

**Figure 5:**
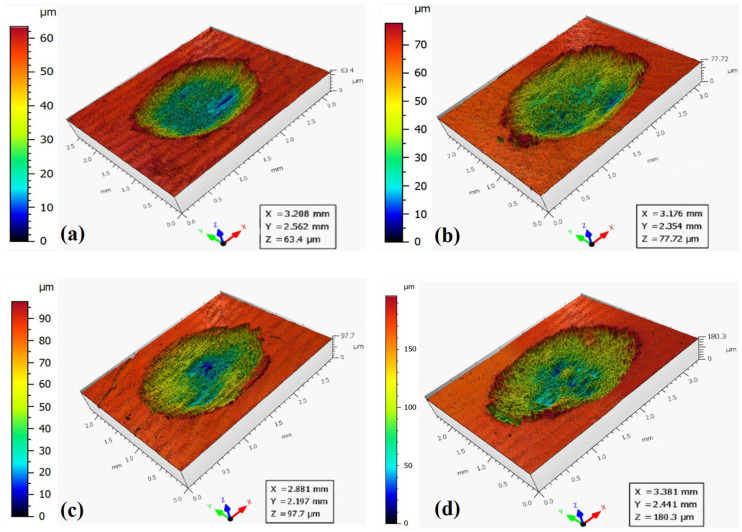
Three-dimensional surface morphology under different applied loads for a displacement of 0.5 mm. (**a**) 20 N. (**b**) 50 N. (**c**) 100 N. (**d**) 200 N.

**Figure 6:**
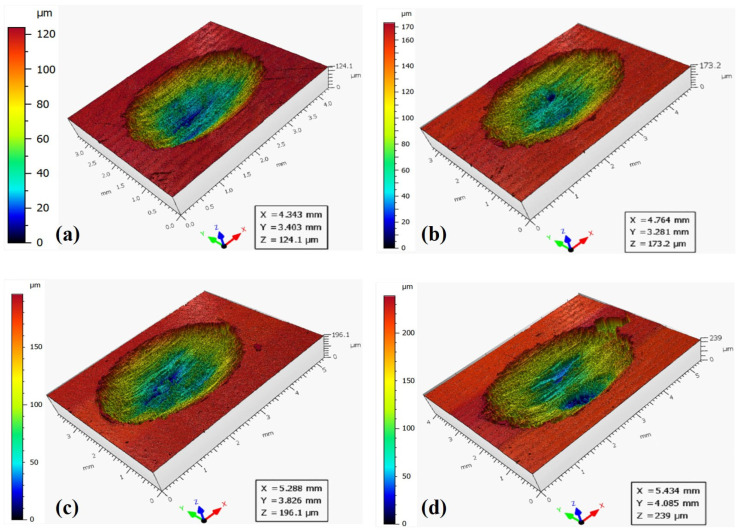
Three-dimensional surface morphology under different applied loads for a displacement of 1.5 mm. (**a**) 20 N. (**b**) 50 N. (**c**) 100 N. (**d**) 200 N.

**Figure 7:**
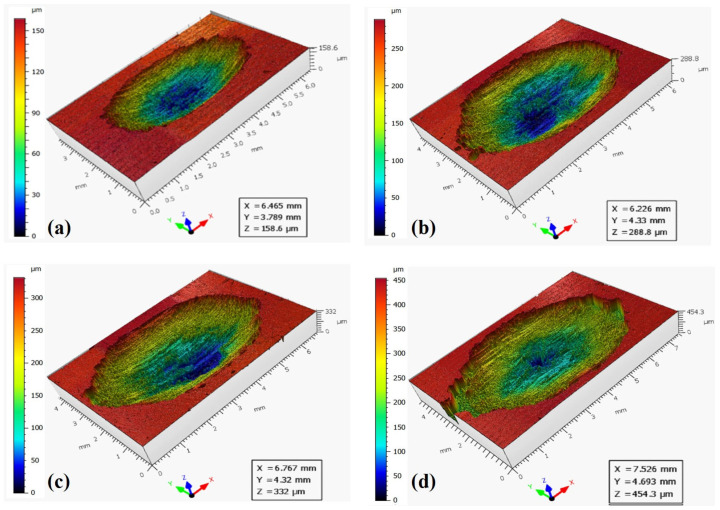
Three-dimensional surface morphology under different applied loads for a displacement of 3.0 mm. (**a)** 20 N. (**b**) 50 N. (**c**) 100 N. (**d**) 200 N.

**Figure 8:**
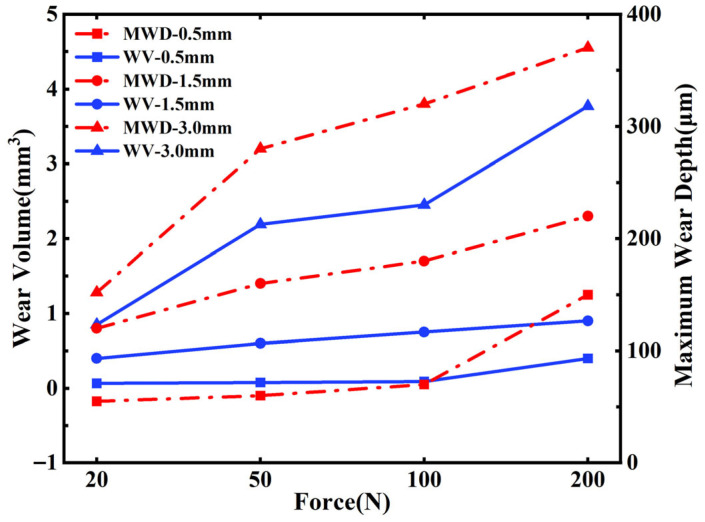
The wear volume and maximum wear depth of AlSi7Mg0.6 aluminum alloy under different displacements and applied loads.

The three-dimensional surface morphologies of wear scars under different displacements and applied loads are shown in Figure 5, Figure 6 and Figure 7. To quantitatively characterize the wear scars, the average wear volume and maximum wear depth were measured repeatedly under different displacements and applied loads and are presented in Figure 8. When the displacement is 0.5 mm, the wear volume shows little variation below 100 N, with a slight increase at 200 N. For a displacement of 1.5 mm, the wear volume increases significantly with applied load. When the displacement is 3.0 mm, the wear volume is much higher than that of the other two displacements. Under the same applied load, the wear volume increases substantially with the displacement, particularly at higher loads. At a displacement of 0.5 mm, the wear volume is approximately 1/10th of that at 3.0 mm. It is also evident that when the displacement is 3.0 mm and the applied load is 20 N, the wear volume is still much greater than that when the displacement is 0.5 mm and the applied load is 200 N. The results indicate that displacement has a more significant effect on wear damage compared to applied load.

## 4. Wear Finite Element Method

### 4.1. Archard Model

In the finite element analysis of the wear evolution of AlSi7Mg0.6 aluminum alloy, it is necessary to establish a friction wear model to calculate the wear depth. The modified Archard wear equation proposed by McColl et al. [17] is adopted in this study, and the Archard model is expressed by Equation (1) [38]:

(1) $$V=KSF_{n}$$

(2) S=2Δxn

where *V* is the wear volume, *F_n_* is the applied load, *S* is the total distance, Δx is the displacement, *n* is the number of wear cycles and *K* is the wear coefficient.

(3) $$h=\frac{V}{A}=\frac{KSF_{n}}{A}$$

The average wear depth can be calculated from the volume of wear, as shown in Equation (3), where *A* is the contact area. In this study, the contact between the steel ball and the aluminum plate is considered a local contact. According to Hertzian contact theory [38], the contact area is represented as shown in Equation (4), where a is the contact radius, $R^{•}$ is the equivalent radius of curvature, and $E^{•}$ is the equivalent elastic modulus [38].

(4) $$A=\pi a^{2}$$

(5) $$a=\sqrt[{3}]{\frac{3F_{n}R^{•}}{4E^{•}}}$$

(6) $$\frac{1}{R^{•}}=\frac{1}{R_{1}}+\frac{1}{R_{2}}$$

(7) $$\frac{1}{E^{•}}=\frac{1-v_{1}^{2}}{E_{1}}+\frac{1-v_{2}^{2}}{E_{2}}$$

where *R*_1_ is the radius of the steel ball. Since the curvature radius *R*_2_ of the aluminum plate approaches infinity, the equivalent radius $R^{•}$ is equal to *R*_1_. *E*_1_ and *v*_1_ are the elastic modulus and Poisson’s ratio of the steel ball, while *E*_2_ and *v*_2_ are the elastic modulus and Poisson’s ratio of the aluminum plate.

Based on Equation (3), the finite element model considers the local relative displacement ds at the contact surface mesh nodes. The wear depth *dh* at each node can be expressed by Equation (8). In the case of wear, as described by McColl et al. [17], Equation (8) can be derived from Equation (9):

(8) $$dh=k_{1}p\left(x\right)ds$$

(9) $$\Delta h\left(x,t\right)=k_{1}p\left(x,t\right)s\left(x,t\right)$$

In the equations, ∆*h*(*x*, *t*), ∆*p*(*x*, *t*), and ∆*s*(*x*, *t*) represent the wear depth, contact pressure, and relative displacement at the contact node during the time increment *t*, respectively, while *k*_1_ denotes the local wear coefficient.

The local wear coefficient *k*_1_ is distinct from the wear volume coefficient *K*, as the latter represents the average value of the wear across the entire wear scar. Unfortunately, it is currently difficult to measure how wear depth changes with contact pressure in experiments, making it impossible to determine the local wear coefficient *k*_1_, and thus it is assumed that both coefficients are set as the same. Since different displacements and applied loads are applied in experiments, the wear coefficient varies for each set of conditions, as shown in Table 2.

Since a single wear cycle in Equation (9) is divided into many small time increments, the finite element simulation requires the product of these increments and the total number of cycles. This results in significant computational time for simulating a complete wear cycle. To address this, this study adopts the cycle-jumping technique proposed by McColl et al. [17] and Mary et al. [19], which assumes that the contact pressure and slip distribution between two surfaces remain the same over the next ∆*N* cycles. Therefore, by multiplying Equation (9) with ∆*N*, Equation (10) is derived, allowing the simulation of the wear corresponding to ∆*N* cycles within a single wear cycle. This method greatly reduces computational time. To ensure consistency in the number of wear cycles during the simulation, the cycle-jumping values ∆N for three different displacements are set to 300, 100 and 50, respectively.

(10) $$\Delta h\left(x,t\right)=\Delta Nk_{1}p\left(x,t\right)s\left(x,t\right)$$

### 4.2. ALE Adaptive Mesh

**Figure 9:**
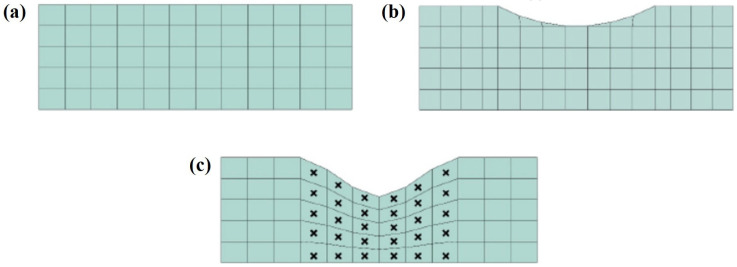
Schematic diagram of ALE adaptive mesh technology. (**a**) Initial mesh. (**b**) Mesh after wear without ALE technology. (**c**) Mesh after wear with ALE technology.

For simulating nonlinear large deformation problems such as friction and wear, ABAQUS provides ALE adaptive mesh technology. This method combines both Lagrangian and Eulerian computational approaches, allowing the model mesh to remain as smooth as possible throughout the computation process. This effectively addresses issues such as mesh distortion and element degeneration caused by excessive mesh deformation. Figure 9 illustrates the working principle of the ALE adaptive mesh technique. In Figure 9a, the initial, complete mesh models are shown. Figure 9b shows the phenomenon of significant deformation of the contact surface without using the UMESHMOTION subroutine, resulting in deformation and distortion of the mesh. In contrast, Figure 9c shows the mesh model after wear, where the UMESHMOTION subroutine is used in conjunction with the ALE adaptive mesh technique (the regions marked with symbols represent areas where the ALE mesh elements have been redefined). As shown in the figures, under the influence of the ALE adaptive mesh technique, the mesh becomes smoother compared to the case without this method, which is more conducive to simulating friction and wear.

### 4.3. Finite Element Model

**Figure 10:**
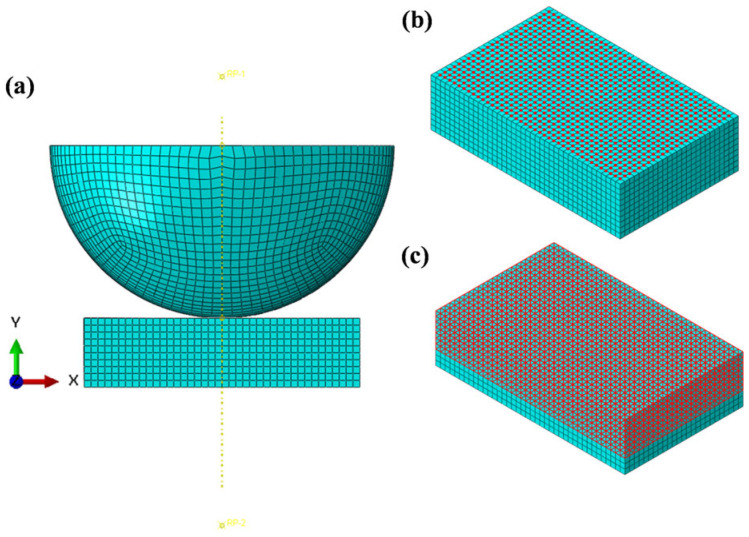
Finite element mesh of GCr15 steel ball and AlSi7Mg0.6 aluminum alloy. (**a**) Geometric mesh model. (**b**) ALE-controlled nodes. (**c**) ALE-defined element regions.

To save computational time, the model is appropriately simplified. The GCr15 steel ball with a diameter of 10 mm is simplified as a hemisphere in the calculations. Based on experimental results, the maximum wear area length does not exceed 8 mm, and the width of wear area is less than 5 mm. Therefore, the AlSi7Mg0.6 aluminum alloy sample, with dimensions of 20 mm × 15 mm × 4 mm, is simplified to a geometry of 8 mm × 5 mm × 2 mm in the finite element analysis. The contact area between the two materials is meshed with finer elements, and the mesh element type adopted is hexahedral C3D8R, as shown in Figure 10a. For the convenience of applying loads and boundary conditions, the upper and lower cross-sections are coupled using reference points RP1 and RP2 to control their motion states. RP2 is fully fixed, while RP1 applies the load in the first step and the displacement in the second step for wear finite element analysis. The regions controlled by the ALE adaptive mesh, including nodes and elements, are shown in Figure 10b,c.

The material properties used for the finite element analysis are shown in Table 3. In the wear simulation process, the material’s density can be neglected. However, to measure the volume change after wear, the material’s density needs to be included.

**Figure 11:**
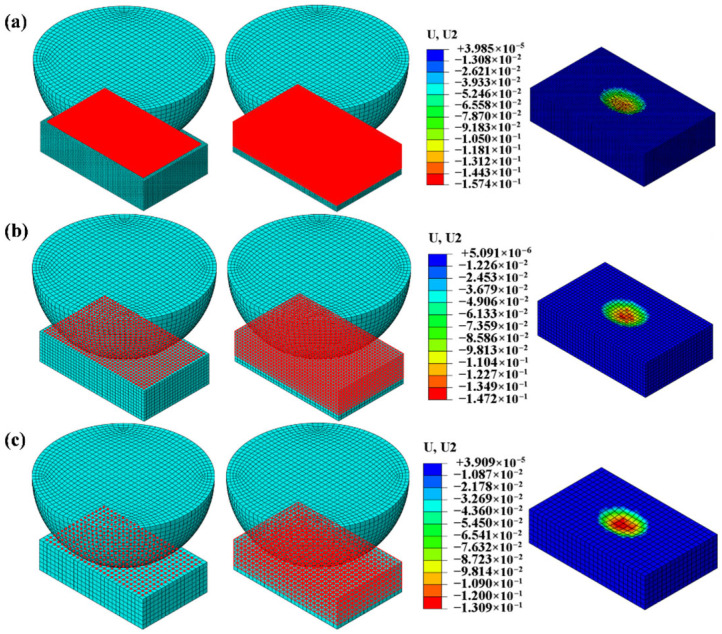
Aluminum alloy plate models with three different mesh sizes. (**a**) 0.1 mm. (**b**) 0.2 mm. (**c**) 0.3 mm.

Simulations were conducted using mesh sizes of 0.1 mm, 0.2 mm, and 0.3 mm to model the wear morphology of aluminum alloy under a 200 N load with a displacement of 0.5 mm, as shown in Figure 11. Under this loading condition, the maximum measured wear depth in experiments was 151.2 μm, with a wear volume of 0.4 mm^3^. Comparing the MWD and WV of aluminum alloys across the three mesh sizes (as shown in Table 4), the 0.2 mm mesh size was selected for wear simulation in this study after comprehensive consideration of computational efficiency and accuracy.

## 5. Results and Discussion

**Figure 12:**
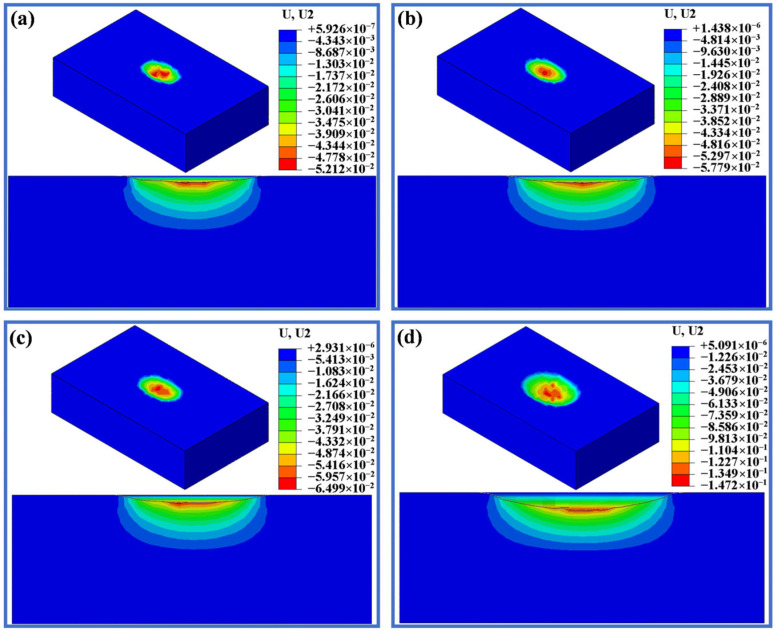
Simulated wear scars with a displacement of 0.5 mm under different applied loads. (**a**) 20 N. (**b**) 50 N. (**c**) 100 N. (**d**) 200 N.

**Figure 13:**
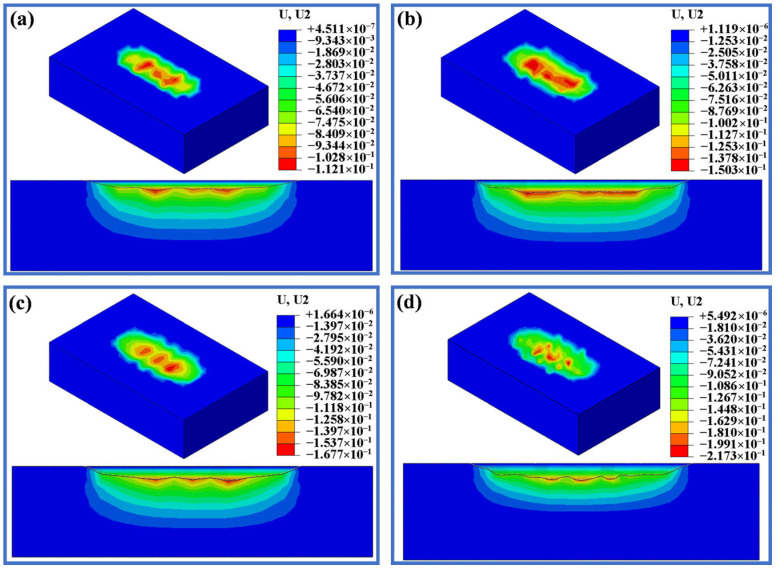
Simulated wear scars with a displacement of 1.5 mm under different applied loads. (**a**) 20 N. (**b**) 50 N. (**c**) 100 N. (**d**) 200 N.

**Figure 14:**
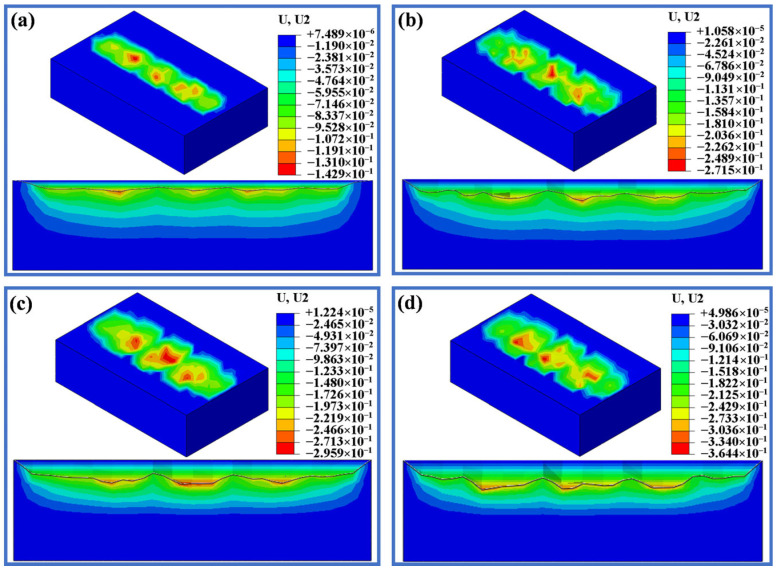
Simulated wear scars with a displacement of 3.0 mm under different applied loads. (**a**) 20 N. (**b**) 50 N. (**c**) 100 N. (**d**) 200 N.

Figure 12, Figure 13 and Figure 14 display the deformation of three different displacements after wear under loads of 20 N, 50 N, 100 N and 200 N. As shown in Figure 12, when the displacement is 0.5 mm, the wear depth and scar changes are minimal under loads of 20 N, 50 N and 100 N. When the load is increased to 100 N, the maximum wear depth only increases by 13 µm compared to 20 N, but there is a sharp increase in wear depth when the load reaches 200 N. Figure 13 and Figure 14 display the wear scars obtained from the finite element analysis. When the displacement is 1.5 mm and 3.0 mm, the wear scars are rough and uneven, while the wear scars for the displacement of 0.5 mm show a smoother surface. It is seen from Figure 13 that when the displacement is 1.5 mm, the wear depth increases slightly with increasing load, but when the load reaches 200 N, the maximum wear depth reaches 217 µm, which is significantly larger. As seen in Figure 14, with a displacement of 3.0 mm, both the wear width and depth significantly increase compared to a displacement of 1.5 mm. The maximum wear depth at 50 N shows a sharp increase of 129 µm compared to the 20 N load, while the increase from 50 N to 100 N is only 24 µm. At a load of 200 N, the wear depth reached a maximum of 364.4 μm among the 12 conditions.

**Figure 15:**
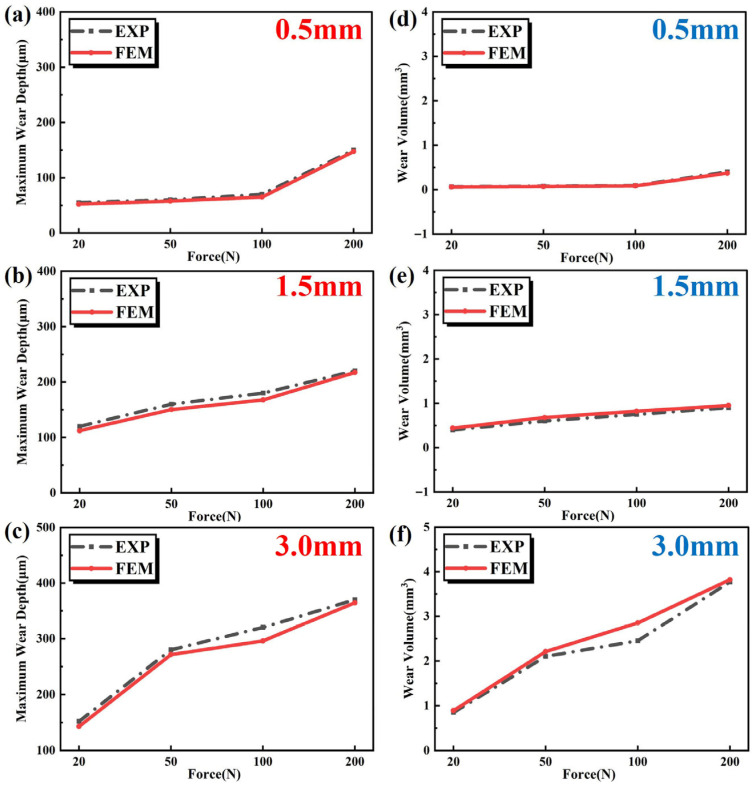
Comparison of maximum wear depth and wear volume between FEM and experimental results, where black line represents experimental results and red line represents simulation results. (**a**) maximum wear depth under sliding displacement of 0.5 mm, (**b**) maximum wear depth under sliding displacement of 1.5 mm, (**c**) maximum wear depth under sliding displacement of 3.0 mm, (**d**) wear volume under sliding displacement of 0.5 mm, (**e**) wear volume under sliding displacement of 1.5 mm, (**f**) wear volume under sliding displacement of 3.0 mm.

The maximum wear depths obtained via FEM are plotted alongside experimental results in Figure 15a–c. Using the volume measurement tool provided by ABAQUS, the volume change of components before and after wear is measured, and the wear volume of AlSi7Mg0.6 aluminum alloy is obtained and compared with experimental wear volume, as shown in Figure 15d–f. From Figure 15a,d, it is observed that the FEM and experimental results for maximum wear depth and wear volume follow a good correlation with the change in load when the displacement is 0.5 mm. For the cases with a displacement of 1.5 mm, the comparison under different loads between FEM and experimental results is shown in Figure 15b,e. It is evident that at 100 N, the maximum wear depth differs most from the experimental result, while at the maximum load of 200 N, the difference is smallest. The largest discrepancy in wear volume occurs under a load of 50 N. From Figure 15c,f, it can be concluded that when the displacement is 3.0 mm, the maximum wear depth and wear volume at 100 N show the largest discrepancy with experimental results, whereas the smallest difference is observed at 200 N. The finite element wear model developed in this study is based on initially idealized smooth surfaces and constant material properties; therefore, the discrepancies between the FEM predictions and the experimental wear characteristics mainly originate from the following two aspects. (1) The friction and wear experiments were conducted in dry air, where an ultrathin oxide film can rapidly form on the aluminum alloy surface. During repeated sliding, this oxide layer continuously forms and spalls off, thereby affecting the wear rate of the aluminum alloy. Such environment-mediated, time-dependent chemo-mechanical coupling effects are not accounted for in the current purely mechanical wear model. (2) The wear debris generated during the experiments is not immediately removed but instead participates in the contact region to form a dynamic mechanically mixed layer. The evolving mechanical properties of this layer continuously alter the friction coefficient and wear mechanism, leading to deviations between the experimentally measured wear characteristics and the predictions of the model based on direct contact between pristine materials.

**Figure 16:**
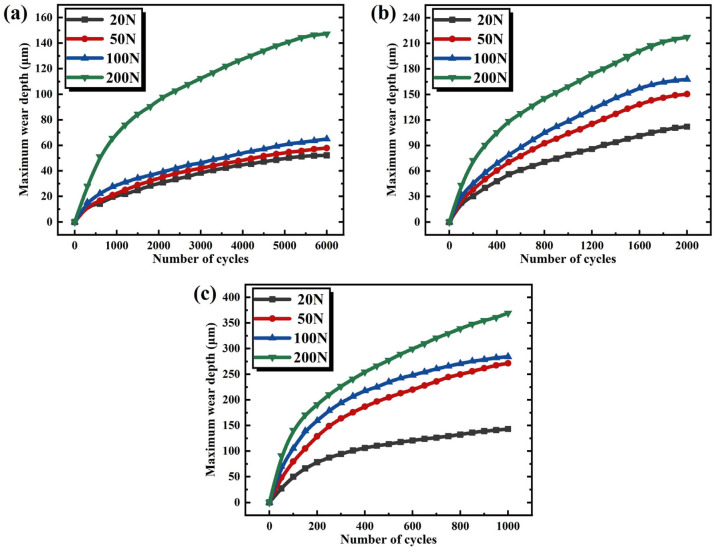
The curves of maximum wear depth varying with the number of wear cycles at different displacements. (**a**) 0.5 mm. (**b**) 1.5 mm. (**c**) 3.0 mm.

Figure 16 illustrates the variation curves of maximum wear depth with increasing wear cycles under different loads for three displacements. All three groups exhibit identical trends: during the initial wear stage, the curves demonstrate a relatively steep upward trajectory, indicating a rapid rate of wear depth progression. As shown in Figure 16a, when wear cycles increase from 0 to 1000, the maximum wear depth under each load displays remarkable growth. This phenomenon occurs because the material surface initially possesses a relatively rough microstructure, resulting in smaller actual contact areas during interaction. Consequently, pronounced local stress concentration significantly accelerates wear rates, enabling rapid wear depth accumulation within a short period. As wear cycles further increase, continuous friction progressively smoothens the material surface, enlarging the contact area and promoting more uniform stress distribution. Thus, the wear depth progression rate decelerates during the intermediate stage. Comparing wear depth variations under different loads (e.g., Figure 16b), the slope of the maximum wear depth curve under 200 N load is markedly steeper than that under 20 N during the initial 600 cycles. This discrepancy stems from higher loads inducing greater surface stress, rendering the material more susceptible to elastic deformation and fatigue wear, thereby accelerating overall wear rates and wear depth progression. In Figure 16c, the curve for a 3.0 mm displacement exhibits an even steeper ascent. This is attributed to longer displacement subjecting the material surface to extended friction paths, leading to more frequent frictional interactions per unit time and faster accumulation of frictional work. Consequently, wear depth increases at an accelerated pace, manifesting as a more pronounced upward trend in the curve.

**Figure 17:**
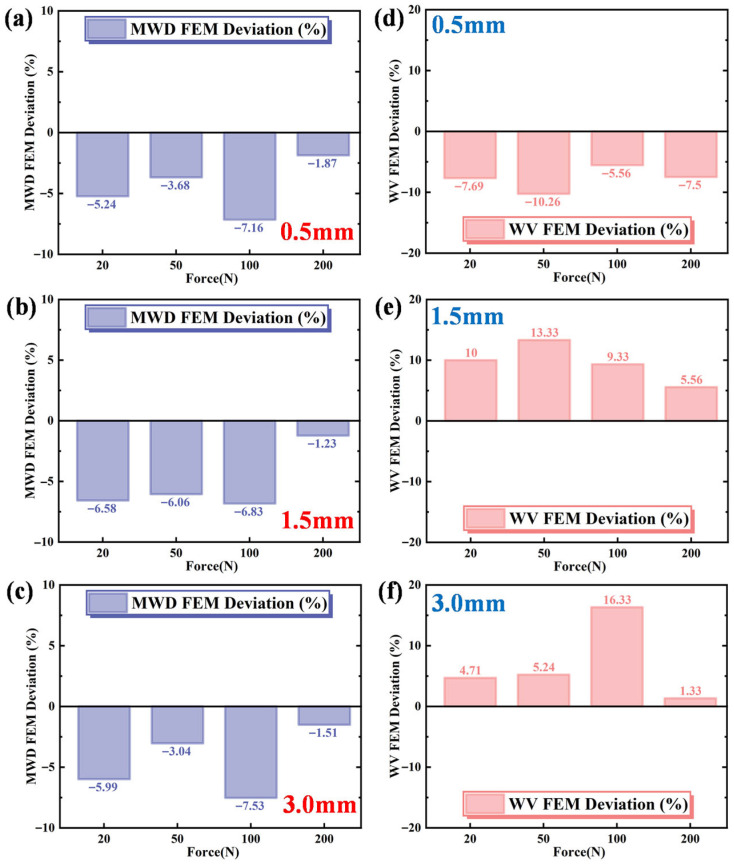
Deviation between FEM and experimental results for maximum wear depth (MWD) and wear volume (WV). (**a**) maximum wear depth under sliding displacement of 0.5 mm, (**b**) maximum wear depth under sliding displacement of 1.5 mm, (**c**) maximum wear depth under sliding displacement of 3.0 mm, (**d**) wear volume under sliding displacement of 0.5 mm, (**e**) wear volume under sliding displacement of 1.5 mm, (**f**) wear volume under sliding displacement of 3.0 mm.

To better illustrate the differences between the FEM and experimental results, the maximum wear depth (MWD) and wear volume (WV) are plotted in terms of deviations in Figure 17. From the figure, it is seen that the maximum wear depth under all three displacements and different loads has a deviation of less than 10% compared to experimental results. Although the deviation in wear volume may be larger in some cases, it does not exceed 20%. Specifically, for a displacement of 0.5 mm, the FEM values are consistently lower than the experimental values. For displacements of 1.5 mm and 3.0 mm, the maximum wear depths in the simulation are smaller than those in the experiments, but the wear volumes are larger in simulations. Therefore, sliding contact friction results in larger discrepancies compared to rolling contact wear. In comparison to the deviation in maximum wear depth, the deviation in wear volume is relatively larger. The inherent limitations of the Archard wear model led to relatively large deviations in the prediction of aluminum alloy wear characteristics under high-displacement and high-load conditions, which can mainly be attributed to the following three factors. (1) The wear coefficient *K* is assumed to be constant, thereby neglecting its dynamic evolution during the wear process. (2) The effects of abrasive debris generated during sliding are not considered. In the present model, wear evolution is realized by updating finite element mesh nodes, without accounting for the formation of a debris-induced protective layer on the worn surface, which can reduce the wear rate of the aluminum alloy. (3) The cycle jump technique assumes that the contact pressure and slip distributions remain unchanged over multiple cycles (Δ*N*). While this assumption is valid for mild steady-state wear, its applicability decreases under high-displacement and high-load conditions.

## 6. Conclusions

The effects of displacement and applied load on the wear performances of AlSi7Mg0.6 aluminum alloy were investigated in this study and the main results are summarized as follows:

(1) Experimental results indicate that at the same displacement, both the maximum wear depth (MWD) and wear volume (WV) of the aluminum alloy increase with rising load. Under identical load conditions, the greater the displacement, the more pronounced the changes in the aluminum alloy’s MWD and WV become.
(2) Based on Archard’s wear theory, a UMESHMOTION user-defined subroutine was developed using FORTRAN programming, and combined with the ALE adaptive meshing technology in ABAQUS, a finite element analysis was conducted to simulate the wear evolution of AlSi7Mg0.6 aluminum alloy in contact with GCr15 steel balls under different displacements and applied load conditions. Wear performances under three different displacements (0.5 mm, 1.5 mm, 3.0 mm) and four different loads (20 N, 50 N, 100 N, 200 N) were carried out, where the maximum wear depth and wear volume were investigated in detail. It is shown that under a displacement of 0.5 mm, the wear depth demonstrated no significant difference under different loads, while under a displacement of 3.0 mm, the wear depth exhibited rupture increase with increased loads. In addition, it was also shown that the wear depth and wear volume under the case of 0.5 mm and 200 N were very close to those under the case of 3.0 mm and 20 N, which was also evidenced by the experiments.
(3) The FEM results show that under three different displacements and four different loads, the deviation between the simulated maximum wear depth and the experimental results does not exceed 10%. While the deviation in wear volume fluctuates more significantly, it remains below 20%. Comparison with experimental results demonstrates that the proposed numerical method can accurately predict wear depth and wear volume under different displacements and loads. Therefore, under more severe wear conditions, the proposed FEM-based approach can be applied to predict the wear behavior of railway catenary components manufactured with AlSi7Mg0.6 aluminum alloy, providing a useful reference for routine inspection and maintenance of railway systems.

## Figures and Tables

**Table 1:** Chemical composition of the AlSi7Mg0.6 alloy samples (wt.%) [37].

Si	Fe	Cu	Mn	Mg	Al	Zn	Ti	Others
7.446	0.157	0.004	0.002	0.655	91.51	0.065	0.151	0.01

**Table 2:** Wear coefficient *K* under different loading conditions.

Displacement	0.5 mm				1.5 mm				3.0 mm			
Applied load (N)	20	50	100	200	20	50	100	200	20	50	100	200
Wear coefficient *K* (×10^−7^)	2.71	1.30	0.75	1.67	16.7	1.00	6.25	3.75	35.4	35.0	20.4	15.7

**Table 3:** Material properties.

Materials	Elastic Modulus (GPa)	Poisson’s Ratio	Density (kg/m^3^)
GCr15 steel ball	210	0.3	7800
AlSi7Mg0.6 aluminum alloy	70	0.33	2610

**Table 4:** Mesh size sensitivity analysis.

Mesh Size	0.1 mm	0.2 mm	0.3 mm
Mesh node	86,751	11,726	4032
Mesh element	80,000	10,000	3213
MWD	157.4 μm	147.2 μm	130.9 μm
WV	0.47 mm^3^	0.47 mm^3^	0.47 mm^3^
Computation time	20 h	8 h	3.5 h

## Data Availability

The original contributions presented in this study are included in the article. Further inquiries can be directed to the corresponding author.
